# Announcement: Sixteenth Conference on Coordination Chemistry

**DOI:** 10.1155/MBD.1996.115

**Published:** 1996


					ON
SIXTEENTH CONFERENCE
COORDINATION CHEMISTRY
PROGRESS IN COORDINATION AND ORGANOMETALLIC CHEMISTRY
The Organizing Committee invites you to participate in the 16th Conference on Coordination Chemistry. The
Conference will be organized by the Slovak Chemical Society, Slovak Technical University and Comenius
University in Bratislava and held in Smolenice Castle from
June 9 to June13, 1997
LOCATION
For more thn thirty years the international conferences on coordination chemistry have regularly been
organized in Slovakia. The Conferences took place in Smolenice Castle that is owned by the Slovak Academy
of Sciences.
The Castle is situated in the middle of forests and parks on the southern slopes of the Low Carpathians. It is a
wonderful place with a quiet atmosphere that creates ideal preconditions for fruitful and useful discussions.
As a rule, the weather in the region is very pleasant in the first days of June, with temperatures of 20-25 C.
Since the Castle is about 60 km far from Bratislava, bus transport from Bratislava to Smolenice and return will
be organized on arrival and departure days, respectively. Bratislava can be reached by plane, train, bus (from
Vienna or Prague) or ship (from Vienna).
LANGUAGE
The official language of the Conference will be English. No interpreting will be offered.
SCOPE OF THE CONFERENCE
With regard to the tradition, the Conference will deal with up-to-date problems of inorganic, coordination and
organometallic chemistry and the progress achieved in these fields. The scientific programme will be
concentrated around the following themes:
A Synthesis, structure and properties of coordination and organometallic compounds
B Reactivity and stability of coordination and organometallic compounds
(catalysis, photochemistry, mechanisms, etc.)
C Complexes in biological systems and human medicine
D Applied and environmental inorganic and coordination chemistry
SCIENTIFIC PROGRAMME
The scientific programme is supposed to be divided into four working days. Each of the topics will be
introduced by a plenary lecture (45 minutes) delivered by an invited lecturer.
The other participants may present their contributions to the scientific programme of the Conference as:
plenary lectures lasting 30 minutes, discussion included,
section lectures lasting 15 minutes, discussion included.
115
However, each participant can deliver only one lecture and thus, the applicants are kindly asked to indicate in
Preliminary Applications their wishes concerning presentation of the lectures. Neveltheless, the final decision
is left to the Organizing Committee.
Remember please, that no posters will be presented at the Conference.
CONTRIBUTED PAPERS
Each participant is expected to submit one paper only. Abstracts of all papers will be published in the Book of
Abstracts of the 16th Conference on Coordination Chemistry.
Moreover, all papers will be reviewed by referees and most of them will be published in the
extent of six pages in a monograph entitled "Progress in Coordination and Organometallic Chemistry" that will
be printed by the SIovak Technical University Press in Bratislava. Naturally, editors will take into
consideration the recommendations of the referees.
The participants will receive the Book of Abstracts as well as the monograph together with reprints of their
papers still before the Conference at the registration.
(Further details in the Second Circular)
SOCIAL PROGRAMME
The social programme, including a welcome party and final banquet, is planned. Besides that, a half-day
optional excursion (cultural and social event) in the surroundings of Smolenice will be organized.
(More details in the Second Circular)
ACCOMMODATION
Accommodation for all participants will be arranged in Smolenice Castle. Since there are only 90 beds
available for participants in the Castle, the number of participants is obviously limited by this number.
(Further information in the Second Circular.)
FEES
1) The registration fee is US $ 150.
This sum will cover all expenses connected with:
monograph, book of abstracts, reprints, conference materials, services.
2) The Conference fee is supposed to be not higher than US $ 150.
It includes all expenses for:
board in Smolenice Castle (three complete meals per day, coffee&tea), social programme, services during
the Conference, travel expenses from Bratislava to Smolenice and return.
3) The lodging in Smolenice Castle will be paid separately.
Thanks to the sponsorship of the SIovak Academy of Sciences, the sum paid for five nights should not
exceed US $ 200 (depending on the quality of rooms).
PRELIMINARY APPLICATION
Enclosed you will find a Preliminary Application Form. Please complete and retum it as soon as possible but it
must reach the Organizing Committee before May 31st, 1996
The Organizing Committee has no objections if you make some more copies of the Preliminary Application
Form for the case that your coworkers would like to apply.
SECOND CIRCULAR
The Second Circular will be distributed at the end of October 1996.
CORRESPONDENCE
All correspondence conceming the 16th Conference on Coordination Chemistry should be addressed to the
chairman of the Organizing Committee:
Professor Gregor ONDREJOVIC
Department of Inorganic Chemistry
SIovak Technical University
Radlinskeho 9,
81237 BRATISLAVA SIovakia
E-mail"
Telephone: ++427495257
Fax:++427493198
sirota @ cvtstu.vt.stuba.sk
116

				

